# High-frequency rTMS improves quality of life and depressive symptoms in Parkinson’s disease: A case report

**DOI:** 10.1016/j.heliyon.2022.e12196

**Published:** 2022-12-10

**Authors:** Panayiota Michael, Sandra Blythin Constantinou Juhasz, Olympia Evagorou, Lilia Psalta, Georgios Mikellides

**Affiliations:** a Cyprus rTMS Centre, Cyprus; b Department of Psychology, Education and Child Studies, Erasmus University Rotterdam, the Netherlands; c Department of Psychiatry, Medical School, Democritus University of Thrace, Greece; d Department of Psychology, University of Cyprus, Cyprus; e School of Science, University of Central Lancashire, Cyprus; f Department of Cognitive Neuroscience, Faculty of Psychology and Neuroscience, Maastricht University, the Netherlands; g Medical School, University of Nicosia, Cyprus

**Keywords:** Parkinson’s disease, Repetitive transcranial magnetic stimulation, High-frequency, Quality of life, Motor symptoms, Non-motor symptoms, Depressive symptoms

## Abstract

**Introduction:**

Parkinson's disease (PD) is a common neurodegenerative disorder, characterised by both motor and nonmotor symptoms. There is currently no cure for PD, although there are several treatment options for relieving PD symptoms. Repetitive transcranial magnetic stimulation (rTMS) is a noninvasive brain stimulation therapy that shows promising results for the treatment of PD.

**Methods:**

Here, we present a patient with PD. We investigated whether an accelerate form of high-frequency (HF) rTMS on the contralateral side to the patient’s main difficulties is clinically effective in treating health-related quality of life (QoL) symptomatology and depressive symptoms in PD as well as the long-term effects of rTMS in PD during the maintenance phase.

**Results:**

Results showed that HF-rTMS administered over the right primary motor cortex (M1) is a safe and well-tolerated treatment that improved the patient’s health related QoL and depressive symptoms. These positive effects lasted at least five months post treatment.

**Conclusion:**

Therefore, HF-rTMS over the right M1 can be a possible treatment option for patients with PD, although further investigations are necessary to validate the findings of the present case report.

## 1 Introduction

Parkinson’s disease (PD) is the most common movement disorder and second most common neurodegenerative disorder [1], characterised by both motor and nonmotor symptoms (NMS). It is prevalent in approximately 1% of the population over 60 and 3–4% of the population over 80 years old [1, 2]. The term *parkinsonism* is used to describe the motor symptoms of PD: tremors, muscle rigidity, and depressed movement [3, 4]. The most prevalent NMS include, among others, depression, anxiety, psychosis, sleep disturbances, autonomic dysfunction, and dementia [4, 5, 6, 7]. The impact of NMS on quality of life (QoL) is evident through the international study by Martinez-Martin et al. [8] who identified an average of 9–12 NMS per patient in their sample of 545 individuals. Also, the high comorbidity of anxiety and depression in PD has even led researchers to suggest that both are regarded as preliminary symptoms for PD [9, 10, 11, 12].

Pharmacological treatments for NMS include the use of antidepressants, antipsychotics, and anxiolytics for comorbid mood disorders [9, 10]. However, medications used for treating motor and NMS of PD can interact and often exacerbate symptomology. Deep-brain stimulation (DBS) is an approved nonpharmacological treatment that stimulates brain regions via electrical impulses [13] and has been beneficial in treating PD symptomology [14]. Although the exact mechanisms of DBS are unknown [15], there is compelling evidence for its effective alleviation of rigidity, dyskinesia, and tremors [16]. However, evidence for the effectiveness of DBS in treating NMS is dubious [15, 17, 18].

Over the past two decades, interest increased on the less invasive procedure of repetitive transcranial magnetic stimulation (rTMS) for treating PD [15]. With rTMS, high currents of single, repeated magnetic pulses are delivered to the targeted brain region at high or low frequencies, lowering motor cortex and cortical activity respectively [19, 20]. Low frequency rTMS (LF-rTMS) administers magnetic pulses of ≤1Hz that have inhibitory effects on cortical excitation, while high frequency rTMS (HF-rTMS) (≥5Hz) increases cortical excitation [19, 21]. Several studies investigated the effects of rTMS on PD motor and nonmotor symptoms. HF-rTMS has been found to positively influence voice and speech [22] and depression [23] in PD, while LF-rTMS has been shown to alleviate parkinsonism [24, 25]. Such findings are crucial, given that alleviating motor and mood symptoms in PD could improve patients’ QoL. However, while rTMS shows promising results in treating motor and nonmotor PD symptoms, research findings are inconclusive, lack uniformity in their methods of conducting rTMS, and placebo effects cannot be ruled out [21, 26]. In this single casereport, we describe the effects of HF-rTMS over the right primary motor cortex in a patient suffering from PD. We wanted to investigate whether an accelerate form of HF-rTMS, on the contralateral side to the patient’s main difficulties, is clinically effective in treating health-related QoL symptomatology and depressive symptoms in PD. Furthermore, we investigated the long-term effects of rTMS in PD during the maintenance phase.

## 2 Case report/case presentation

### 2.1 Participant

**Figure 1:**
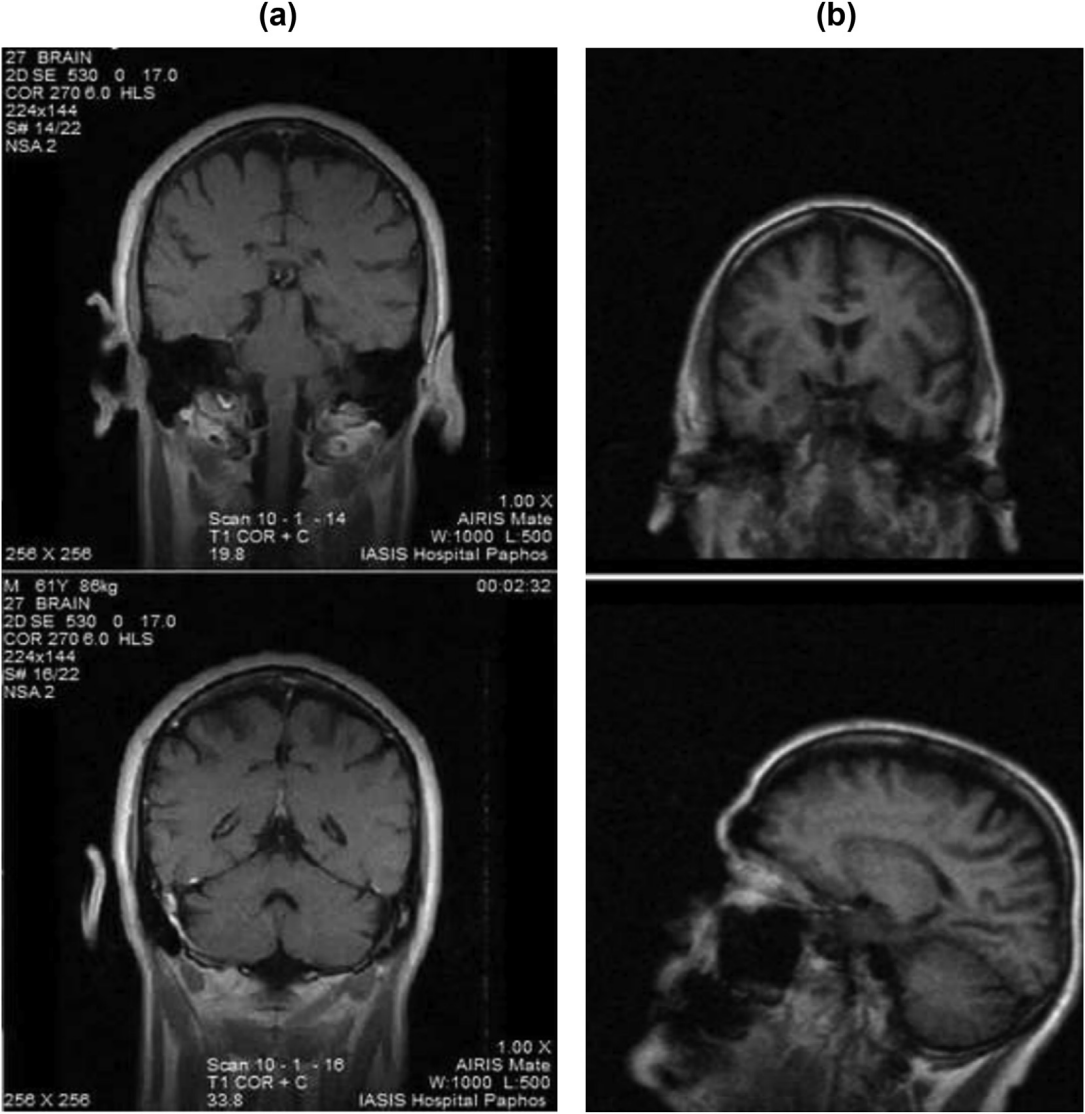
(A) Coronal sections of magnetic resonance imaging (MRI) performed in 2013, showing brain aging and partially empty sella turcica, (b) Coronal and sagittal sections of MRI performed in 2020, showing mild cerebral atrophy/aging more pronounced in the curvatures of the cerebral hemispheres.

This case study presents a 70-year-old married male (L.P.034) diagnosed with PD. Symptoms were first present in 2013, when the patient reported leg pain. A Magnetic resonance imaging (MRI) scan showed brain aging and partially empty sella turcica with a fine imaging pituitary gland (Figure 1a). Following the MRI scan in 2013, he was diagnosed with PD. During 2020 the patient experienced worsening of his symptoms and had a second MRI scan. The MRI scan showed mild cerebral atrophy/aging more pronounced in the curvatures of the cerebral hemispheres. Also, few, micro-ischemic focal lesions occurred in the periventricular and deep white matter of the cerebral hemispheres (Figure 1b). He initially attended physiotherapy on a daily basis which was subsequently reduced to two or three sessions per week. Following his PD diagnosis, he was prescribed levodopa, entacapone, pramipexole, and rasagiline. Along with PD, the patient experienced comorbid symptoms of depression and anxiety. To treat psychological symptoms, the patient was prescribed escitalopram, amitriptyline hydrochloride, and bromazepam. Solpadine was used to treat pain. Written informed consent for the rTMS treatment and the publication of this paper were obtained from the patient.

### 2.2 Repetitive transcranial magnetic stimulation (rTMS)

The patient underwent rTMS using the MagPro X100 stimulator (MagVenture, Farum, Denmark). Before the first session, the patient’s resting motor threshold (rMT) in the left primary motor cortex (M1) was measured using the Coil C–B60. This determined the intensity required to elicit a motor-evoked potential (MEP) in at least 50% of attempts. The stimulation intensity was set at 100% of the rMT. The rTMS protocol was administrated over the right M1 of the hand using a figure-eight coil (Coil Cool-B65). For locating the targeting area, the 10–20 EEG system was used [27]. The patient received a total of 30 rTMS sessions over a five-week period, where six sessions were administrated per week. Two rTMS sessions were administered per visit with a 40-minute break between sessions. Upon completion of 30 sessions, the patient continued with nine maintenance rTMS sessions. These were scheduled weekly for the first four sessions, biweekly for the fifth and sixth sessions, and once a month for the last three sessions. Each rTMS session was administered according to the following protocol: 10Hz, 25 trains with 40 pulses per train, and 20-second inter-train intervals. This choice follows the bulk of literature which favours HF-rTMS to the M1 for treating motor, and to a lesser extent, depressive symptoms [28]. Due to moderate effect sizes reported in other studies, we have used an accelerated form with more trains and pulses per session, with two administrations of the protocol per session. A total of 2000 pulses were given per session for approximately 20 min.

### 2.3 Clinical assessments

Two self-reported scales were used to assess the health-related QoL in PD (Parkinson’s Disease Quality of Life Questionnaire; PCQ-39) and depression severity (Beck Depression Inventory–II; BDI-II). The PCQ-39 [29] assesses eight factors (Bodily Discomfort, Communication, Cognition, Social Support, Stigma, Emotional well-being, Activities of daily living, Mobility) pertaining to the health-related QoL which can be found in Table 1. PDQ-39 items were rated on a 5-point Likert scale ranging from 0 – 4. The total score and sub-factor scores range between 0 – 100, with 0 indicating the best and 100 the worst QoL [30]. The BDI-II is one of the most widely used multiple-choice self-report instruments, designed to assess depression severity [31]. It consists of 21 items, where each item has a possible score between 0-3. The possible range of the total score is 0–63, with a higher total score indicating more severe depressive symptoms. Specifically, the total scores determine the following classifications: 0–13 are minimal, 14–19 are mild, 20–28 are moderate, and 29–63 are severe depression. For the purposes of the present case, we used the Greek version of PCQ-39 [32] and BDI-II [33]. The patient was assessed on both scales (BDI-II and PCQ-39) at 15 different time points: immediately before the first rTMS session (T0), after six sessions (T1), after 12 sessions (T2), after 18 sessions (T3), after 24 sessions (T4), and after 30 sessions/end of treatment (T5), and then after each maintenance session: one week post treatment (T6), two weeks post treatment (T7), three weeks post treatment (T8), four weeks post treatment (T9), six weeks post treatment (T10), eight weeks post treatment (T11), three months post treatment (T12), four months post treatment (T13), and five months post treatment (T14).

**Table 1:** Sub-factors of PDQ-39 scale.

	Mobility	Activities of daily living	Emotional well-being	Stigma	Social Support	Cognition	Communication	Bodily Discomfort
Treatment								
T0	62.50	37.50	54.17	31.25	16.67	68.75	33.33	58.33
T1	47.50	33.33	37.50	25.00	.00	56.25	33.33	50.00
T2	27.50	37.50	50.00	12.50	.00	43.75	25.00	41.67
T3	25.00	25.00	41.67	12.50	.00	43.75	.00	25.00
T4	27.50	12.50	25.00	12.50	.00	31.25	8.33	25.00
T5	17.50	33.33	50.00	.00	.00	25.00	16.67	25.00
Maintenance								
T6	17.50	20.83	25.00	.00	.00	25.00	16.67	25.00
T7	15.00	16.67	20.83	.00	.00	25.00	16.67	25.00
T8	17.50	12.50	25.00	.00	.00	18.75	8.33	25.00
T9	7.50	12.50	20.83	6.25	.00	12.50	.00	16.67
T10	12.50	16.67	20.83	6.25	.00	25.00	.00	25.00
T11	20.00	16.67	25.00	12.50	.00	25.00	.00	16.67
T12	12.50	20.83	12.50	.00	.00	18.75	.00	25.00
T13	12.50	20.83	16.67	.00	.00	25.00	.00	25.00
T14	15.00	16.67	16.67	.00	.00	18.75	16.67	25.00

Clinical results at the 15 time points: immediately before the first rTMS session (T0), after six sessions (T1), after 12 sessions (T2), after 18 sessions (T3), after 24 sessions (T4), and after 30 sessions/end of treatment (T5), one week post treatment (T6), two weeks post treatment (T7), three weeks post treatment (T8), four weeks post treatment (T9), six weeks post treatment (T10), eight weeks post treatment (T11), three months post treatment (T12), four months post treatment (T13) and five months post treatment (T14).

Additionally, the Mini Mental State Examination (MMSE) was used immediately before the first session (T0) and after the completion of 30 sessions/end of treatment (T5). The MMSE is a 30-point test that is widely used by health-care providers to assess cognitive impairment [34]. Scores of 26 and above indicate normal cognitive functioning, scores between 21-25 indicate mild cognitive impairment, scores between 13-20 indicate moderate cognitive impairment, and scores 12 and below indicates severe cognitive impairment.

## 3 Results

**Figure 2:**
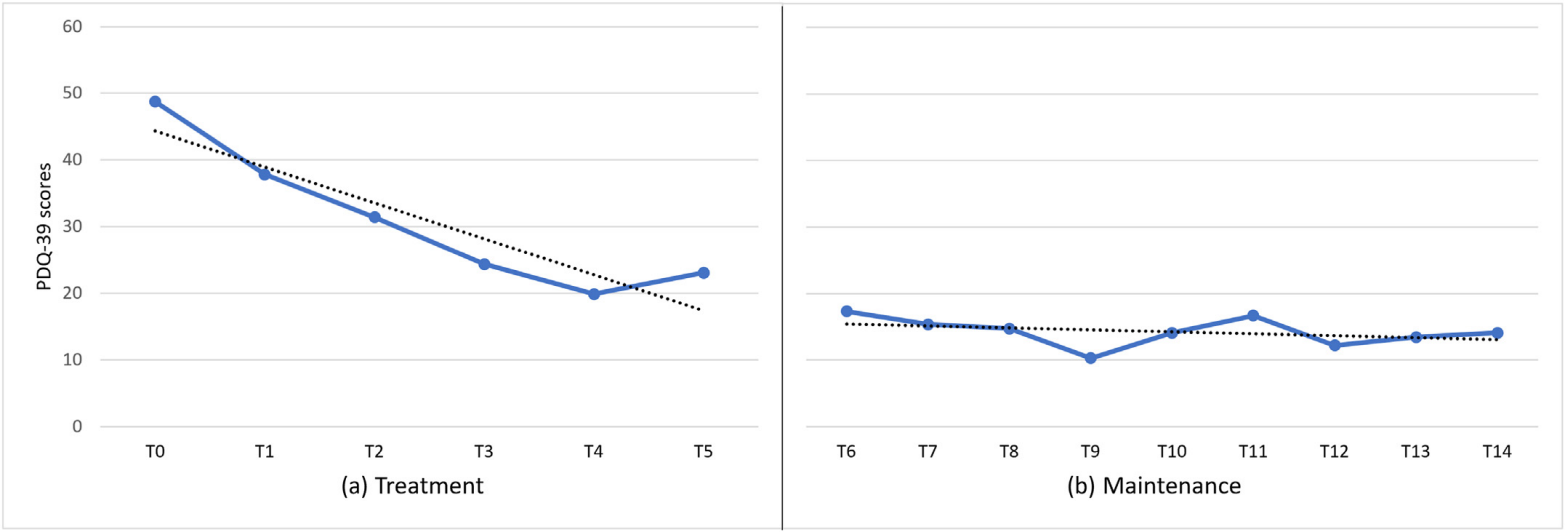
Changes in parkinsonism scores during the treatment and maintenance phases ǀ This line graph shows the difference in PDQ-39 scores at the 15 time points.

**Figure 3:**
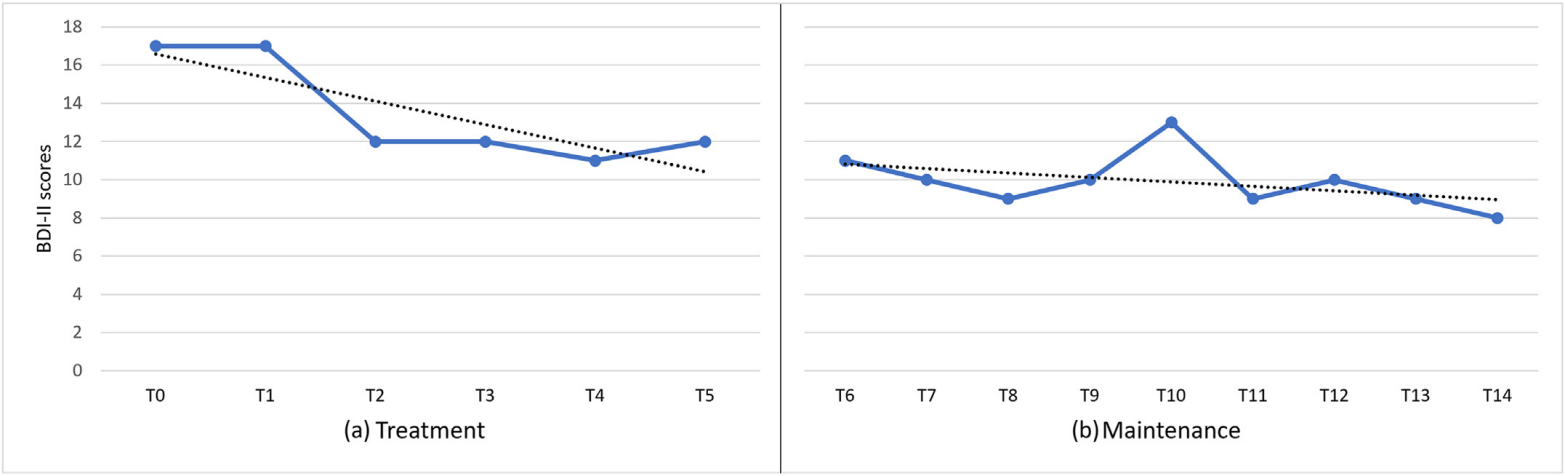
Changes in depression scores during the treatment and maintenance phases ǀ This line graph shows the difference in BDI-II scores at the 15 time points.

No adverse effects of the rTMS were reported throughout the treatment and maintenance periods. The patient completed the treatment and maintenance with observable improvements on both self-reported scales (PDQ-39 and BDI-II). Figure 2 illustrates the changes in parkinsonism scores as measured by the PDQ-39. During the treatment phase, levels range from 48.72 to 19.87 and the trendline indicates that there has been a steady decrease in parkinsonism scores. A slight variation was observed between T4 and T5, where the level increased from 19.87 to 23.08. The level decreased further from the end of treatment (T5) to one week post treatment (T6). During the maintenance phase, levels range from 17.31 to 14.10 and the trend remains approximately stable indicating that there is no change. However, there is a slight variation between T8 and T12. Furthermore, Figure 3 indicates the patient’s reports of depressive symptoms on the BDI-II. During the treatment phase, levels range from 17 to 11. According to the graph, there is a downward trend, which shows that the severity of patients’ depression has decreased from mild to minimal. Nevertheless, the scores fluctuated slightly throughout the treatment period. There is a gently drop from the end of treatment (T5) to one week post treatment (T6). During the maintenance phase, levels range from 11 to 8 and the trendline remains approximately stable with some strong fluctuations between T7 and T11. Specifically, a major variation was observed between T9 and T10, where the level increased from 10 to 13, and then again decreased in T11. After the questionnaire was administered in T10, a discussion followed with the patient, who stated that he was experiencing some temporary personal problems, which may explain this increase. The MMSE score increased to 27/30 after completing 30 sessions (T5) which is indicated as normal compared to 24/30 at the baseline (T0), which indicate mild cognitive impairment.

The patient additionally reported a greater ease in communication. Particularly, he feels more sociable, has less difficulties in conversing, and assists himself more easily. Finally, the patient has reduced his intake of anxiolytics and has terminated physiotherapy subsequent to the rTMS treatment. After completing 30 sessions, the patient underwent physiotherapy again, simultaneously with maintenance. The physiotherapist reported that his left side (the side of the patient's main difficulties) was significantly stronger than before treatment.

## 4 Discussion

In this case study we have found that two administrations of a HF-rTMS protocol per session over the right primary motor cortex is a safe and well-tolerated treatment for PD. These administrations were done on the contralateral side to the patient’s main difficulties. Overall, we have been able to demonstrate that HF-rTMS administered over M1 improved the patient’s health status and QoL, as measured by the PDQ-39. Additionally, the patient showed marked improvements in their depressive symptoms. These results were maintained or further improved during the maintenance phase that lasted up to five months post-treatment. Nevertheless, some fluctuations and slight increases in parkinsonism scores were observed during both phases.

This study’s findings are in accordance with previous research. In the study of Yang et al. [35], it was demonstrated that multi-session HF-rTMS (but not LF-rTMS) over the M1 with a total of 18.000–20.000 pulses was the most efficacious protocol in treating PD. Furthermore, in the review of Lefaucheur et al. [36] advocate for HF-rTMS in the M1 contralateral to the pain side for patients with neuropathic pain and also reported that HF-rTMS of the left dorsolateral prefrontal cortex (DLPFC) can be used to reduce depressive symptoms in PD. Additionally, HF-rTMS of bilateral M1 stimulation [36, 37] and left DLPFC stimulation [37] is likely efficacious in improving parkinsonism symptoms. Another study [38] found support for the effectiveness of bilateral M1 HF-rTMS in treating PD motor symptoms. However, they found no effect of HF-rTMS in the DLPFC on mood, and no added benefit of combined M1 and DLPFC HF-rTMS for improving mood or motor symptoms. In agreement with our study’s findings, a randomised, double-blind, placebo-controlled study by Makkos et al. [39] demonstrated the beneficial effects of bilateral M1 HF-rTMS on depressive symptoms and health related QoL in PD patients with mild or moderate depression. Notably, the antidepressant effects of 10-day HF-rTMS had a lasting effect for up to 30 days.

The importance of post-treatment maintenance should be emphasised. Maintenance of rTMS treatment could prolong and strengthen its antidepressant effects. This, in turn, can delay or even prevent symptom recovery. Studies including patients with treatment resistant depression, have shown that maintenance rTMS can potentially delay relapse after successful treatment [40, 41]. In a more recent randomised, sham-controlled study of maintenance HF-rTMS over the left DLPFC for treatment resistant depression, results indicated that the antidepressant effect of HF-rTMS arose three months post-treatment [42]. Most importantly, maintenance rTMS was well-tolerated and had no side-effects. Finally, a review of rTMS for treatment resistant depression suggested a proposed a treatment protocol of HF-rTMS (10Hz) to the left DLPFC using 3000 pulses per session for a duration of 20–30 sessions [43].

This case study highlights some significant implications. Firstly, HF-rTMS has demonstrated its efficacy in treating motor and affective symptoms of PD. This is particularly important to consider in the case of treatment resistant patients, or patients who experience side effects from medication [44]. RTMS has demonstrated comparable efficacy to pharmacological interventions, bypassing the complications that can arise from medical treatment [44].

Being limited to a single case study, our findings cannot be used to draw scientifically strong and generalisable conclusions. In addition, this study did not include a control or placebo condition. Therefore, future placebo-controlled, double-blinded, randomised trials with powered sample sizes are needed to substantiate the efficacy of HF-rTMS as an alternative for treating motor and mood symptoms of PD. A second limitation to this study is that rTMS was only administered in the M1. Current research consistently shows the benefits of M1 HF-rTMS in treating motor symptoms. However, findings on the effect of HF-rTMS in the M1 and DLPFC in treating PD mood symptoms are conflicting. An interesting line for future research would be to investigate the effect of M1 and DLPFC HF-rTMS on mood symptoms through placebo-controlled, randomised trials.

## 5 Conclusion

rTMS can be a promising alternative treatment for people with Parkinson's disease, taking into account its effectiveness in the motor and NMS of the disease, the lower risk of side effects compared to medication, the improvement in the depressive symptoms, as well as its strengthening effect in medication-treatment-resistance symptomatology.

## Declarations

### Author contribution statement

All authors listed have significantly contributed to the investigation, development and writing of this article.

### Funding statement

This research did not receive any specific grant from funding agencies in the public, commercial, or not-for-profit sectors.

### Data availability statement

Data will be made available on request.

### Declaration of interest’s statement

The authors declare no conflict of interest.

### Additional information

No additional information is available for this paper.
